# The work of the consultation in general practice: a comparison of affluent and deprived areas of Scotland using a novel consultation workload index

**DOI:** 10.3399/BJGPO.2025.0103

**Published:** 2026-05-06

**Authors:** Kieran D Sweeney, Lauren Ng, Stewart W Mercer

**Affiliations:** 1 Usher Institute, College of Medicine and Veterinary Medicine, University of Edinburgh, Edinburgh, UK

**Keywords:** general practice, health inequalities, health inequities, workload index, workload

## Abstract

**Background:**

The workload within GP consultations, and how this varies by deprivation, is not well known.

**Aim:**

To examine how deprivation influences GP consultation workload by developing and applying a novel consultation workload index (CWI).

**Design & setting:**

Secondary analysis of a cross-sectional postal survey of patients who had recently consulted a GP in deprived and affluent areas of Scotland.

**Method:**

The CWI was developed using patient-reported data on: (1) whether more than one problem was discussed in the consultation; (2) whether a complex problem (defined as involving both physical and psychosocial issues) was discussed; and (3) the presence of a disability or limiting long-term condition. Results were analysed by area-level deprivation and consultation modality (face-to-face versus telephone consultations).

**Results:**

Analysis included 721 patients. Correlations between the three variables of the CWI were low (rho<0.2), suggesting that each was capturing a distinct aspect of consultation workload. Using the CWI, more than half of all consultations in deprived areas had ‘high’ (29%) or ‘very high’ (25%) workload, compared with approximately one-quarter in affluent areas (‘high’ 20%, ‘very high’ 6%). This was evident across both face-to-face and telephone consultations.

**Conclusion:**

Greater patient need and complexity in deprived areas is reflected in higher GP workload in the consultation as measured by the CWI. Ways of operationalising the CWI routinely — for example, through real-time artificial intelligence (AI) analysis of consultations — should be explored. If robust, these could be used to inform the resource allocation to general practice to help address the inverse care law.

## How this fits in

Patients in more socioeconomically deprived areas face poorer health than those in affluent areas, but the impact on GP workload within the consultation is not well understood. This study used patient-reported data to develop a novel consultation workload index to quantify GP workload at the level of the consultation. Our findings show that consultations in deprived areas, across both face-to-face and telephone modalities, involve a significantly higher workload compared with affluent areas. These results support the need to incorporate consultation-level workload into resource allocation and workforce planning to enhance support for GPs and patients in deprived areas.

## Introduction

Health inequalities across different socioeconomic populations in the UK are well-documented, with patients in deprived areas experiencing poorer health outcomes compared with those in more affluent areas.^1–3^ These disparities are often described through population-level health outcomes, such as life expectancy or disease prevalence, and system-level demand, such as the number of appointments.^4,5^ However, less is known regarding how these inequalities manifest at the level of the individual consultation.

Consultation workload — which we define as the volume and complexity of tasks in a single patient encounter — is an underexplored workload measure in primary care. It may be influenced by both a patient’s health status and unpredictable consultation-specific factors, including the number and nature of the problem(s) that the patient wants to discuss.^6^ This is of particular relevance in deprived areas, where physical, psychological, and social concerns are more prevalent, resulting in greater complexity and demand at the consultation level, and potentially placing a higher clinical burden on GPs in these settings.^7,8^

Amid the current challenges in UK general practice — marked by increasing patient demand and a declining GP workforce — understanding consultation workload is critical.^9^ Despite its importance for healthcare delivery, evidence on consultation workload differences remains limited, with most research on consultation demand focusing on frequency or duration.^10,11^ The ability to more accurately characterise and examine workload within GP consultations may provide additional insights into how health inequalities affect primary care.

This study aimed to develop and apply a novel consultation workload index (CWI) to assess differences between consultations in deprived and affluent urban areas. It draws on patient-reported data, and is thus a measure of objective workload rather than GPs’ subjective assessment.

## Method

### Study design

This study involved a secondary analysis of data from a cross-sectional survey assessing patients’ experiences of GP consultations in Scotland in 2022.

### Sampling, recruitment, and data collection

Details on design, sampling, and recruitment methods are described in full elsewhere.^6^ In summary, 12 general practices across three health boards in Scotland were purposefully selected to capture a range of geographic and socioeconomic characteristics, and grouped according to whether they served mainly urban–deprived, urban–affluent, or remote and rural areas. In each practice, a random sample of adult patients (aged ≥18 years) who consulted a GP in the prior 30 days was identified from practice records. A total of 6291 surveys were distributed, with 1053 responses received (17% response rate). For the purpose of this secondary analysis study focusing on deprivation, only responses from urban–deprived (*n* = 448, response rate 14%) and urban–affluent (*n* = 273, response rate 27%) areas were included.

### Instruments used

The survey gathered data on patient demographics and health status, including the presence of a disability or limiting long-term illness (*‘Do you have a long-term illness or disability that limits your daily activities?*’), and multimorbidity (using a self-reported checklist of 17 common long-term conditions). Anxiety and depression symptoms were measured using the Patient Health Questionnaire (PHQ-4) scale.^12^ Patients indicated the number and types of problems discussed during their recent consultation with the GP. Problems were classified as complex if they involved both physical and psychosocial elements, or if a physical problem co-existed with anxiety and depression (PHQ-4 score of ≥3), as in previous studies.^2,13,14^ The full patient survey is shown in the supplementary file.

### The consultation workload index (CWI)

A novel CWI was developed using the data from the patient survey, comprising the following three variables:

- Whether the patient had a disability or limiting long-term illness;
- Whether more than one problem was discussed in the consultation; and
- Whether the consultation involved a complex problem.

The concept of the CWI was developed by SWM and further discussed with KS and LN, all of whom are practising GPs. Items (ii) and (iii) were included based on consensus that they directly impact the consultation and, therefore, contribute to workload. The inclusion of item (i) prompted more extensive discussion, particularly regarding whether it should be replaced with a measure of multimorbidity (for example, number of self-reported long-term conditions). While multimorbidity was recognised as a relevant factor influencing consultation complexity, the group noted that its impact was often more pronounced when associated with functional impairment. It was therefore agreed to retain item (i) and to explore the relationship between multimorbidity and disability or limiting long-term illness (hereafter referred to as ‘disability’) as part of this study.

Each of the three variables in the CWI was scored as 0 (not present) or 1 (present), resulting in a total score of 0 (which was assigned as ‘low workload’), 1 (as ‘moderate workload’), 2 (as ‘high workload’), or 3 (as ‘very high workload’). No weightings were applied to any of the three variables.

### Analysis

The relationship between the three variables, and between disability and multimorbidity, was examined by Spearman’s correlation (rho) and by cross-tabs. The CWI was then used to compare consultations in deprived and affluent areas, with further stratification by consultation modality (telephone versus face-to-face consultation). The CWI was treated as missing if any of its three constituent variables were absent, affecting 1% of responses. All analyses were conducted using SPSS (version 27).

## Results

There were 721 responses from 4163 surveys distributed in affluent (*n* = 1000) and deprived (*n* = 3163) areas (17% response rate), with 448 responses from deprived areas (14%) and 273 responses from affluent areas (27%). The characteristics of survey responders are shown in Table 1. Responders in affluent areas had a mean age of 62 years and 55% were female. Responders in deprived areas had a mean age of 61 years and 59% were female. These demographic characteristics did not differ significantly by deprivation, but there were more patients with multimorbidity, disability, complex problems, and more than one problem to discuss in consultations in deprived compared with affluent areas. Significantly more consultations were conducted by telephone in deprived areas (41%), compared with affluent areas (31%) (*P* = 0.003).

**Table 1.** Characteristics of survey responders and their recent consultation, in affluent or deprived areas of Scotland

	Affluent areas *n* = **273**	Deprived areas *n* = **448**	*P* value
**Mean age, years (range)**	62 years (20–95)	61 years (22–92)	0.131
**Sex: *n* (%)**			
Female	149 (55)	266 (59)	0.236
Male	113 (41)	163 (36)	
Other	2 (1)	1 (0)	
Missing	9 (3)	18 (4)	
**Long-term condition count: mean, (SD)**	2.1 (1.6)	3.0 (2.0)	<0.001
**Consultation modality: *n* (%)**			
Telephone	84 (31)	184 (41)	0.003
Face to face	183 (67)	249 (56)	
Other	2 (1)	5 (1)	
Missing	4 (1)	10 (2)	
**Disability or limiting long-term condition: *n* (%)**	68 (25)	251 (57)	<0.001
**>1 problem discussed: *n* (%)**	96 (35)	211 (47)	<0.001
**Complex problem discussed: *n* (%)**	94 (34)	233 (52)	<0.001

### Overlap between the three components of the CWI

The correlations between the three components of the CWI were significant but weak (all rho<0.2) suggesting each variable was capturing a distinct aspect of consultation workload (Supplementary Table S1).

#### Relationship between disability and multimorbidity

The relationship between disability and multimorbidity was also explored, as multimorbidity could potentially be used instead of disability in the CWI. The correlation between disability and multimorbidity count was rho 0.424 (*P*<0.001). Although the chance of having a disability increased with multimorbidity count, there were also many with multimorbidity who did not have a disability (Supplementary Figures S1 and S2).

#### Comparison of workload between affluent and deprived areas

Figure 1 shows the distribution of workload between deprived and affluent areas across all consultations. In deprived areas, 54% of consultations were classified as ‘high’ (29%) or ‘very high’ (25%) workload, compared with 26% in affluent areas (6% ‘very high’, 20% ‘high’). Consultations classified as ‘low workload’ were more common in affluent areas (38%) than in deprived areas (15%). The difference in distribution of consultation workload (low to very high) was statistically significant between the two groups (*P*<0.001).

**Figure 1.**
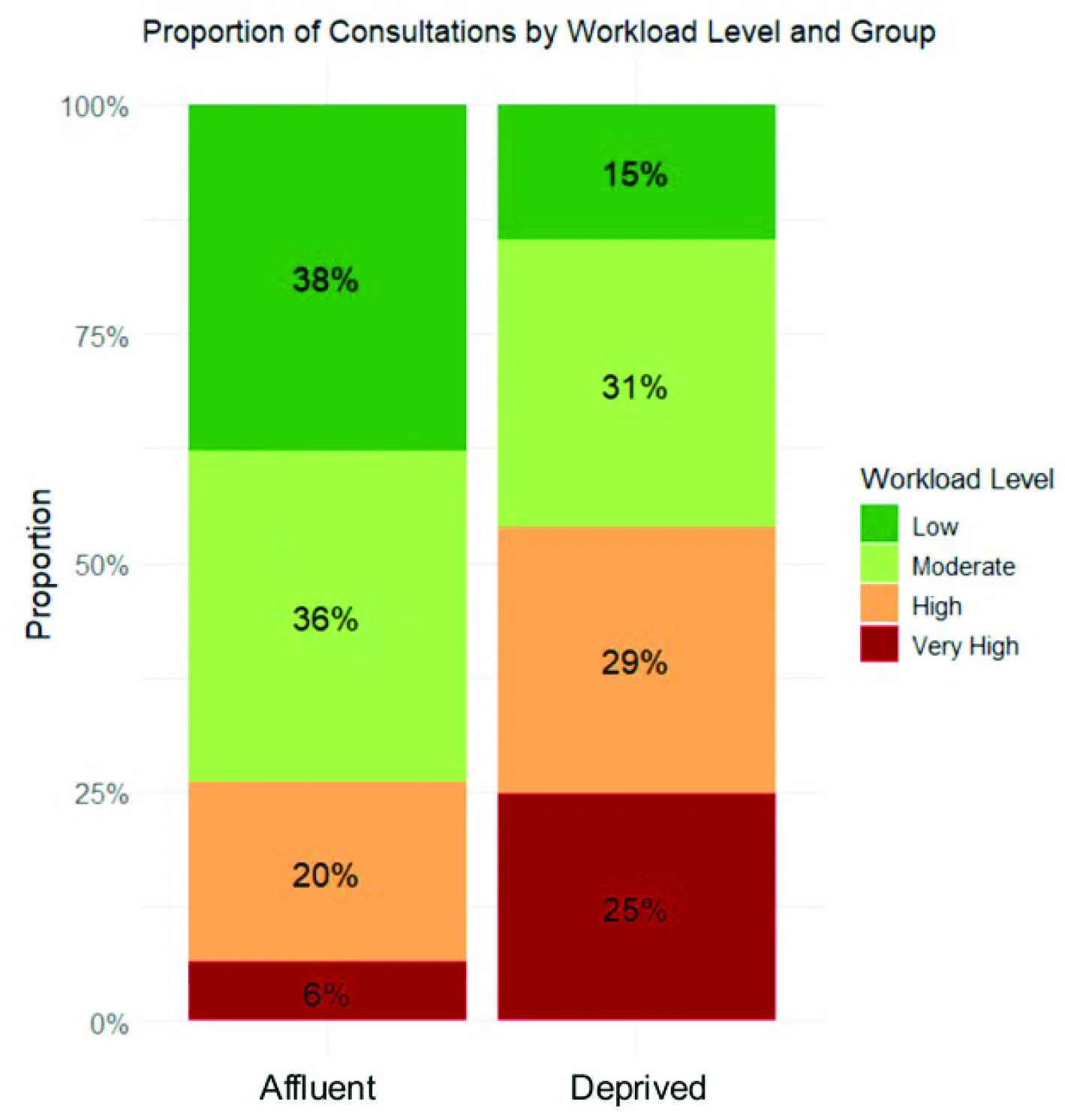
Differences in GP consultation workload between affluent and deprived areas

#### Comparison of workload by consultation modality and socioeconomic group

Figure 2 demonstrates the distribution of consultation workload by consultation modality and socioeconomic group (deprived and affluent). More than half (51%) of telephone consultations in deprived areas had a high or very high workload, compared with 19% in affluent areas. Only 14% of telephone consultations in deprived areas were rated as low workload, compared with 45% in affluent areas. In addition, 58% of face-to-face consultations in deprived areas were high or very high workload, compared with 31% in affluent areas. Face-to-face consultations with a low workload were also less common in deprived areas (13%), compared with affluent areas (33%).

**Figure 2.**
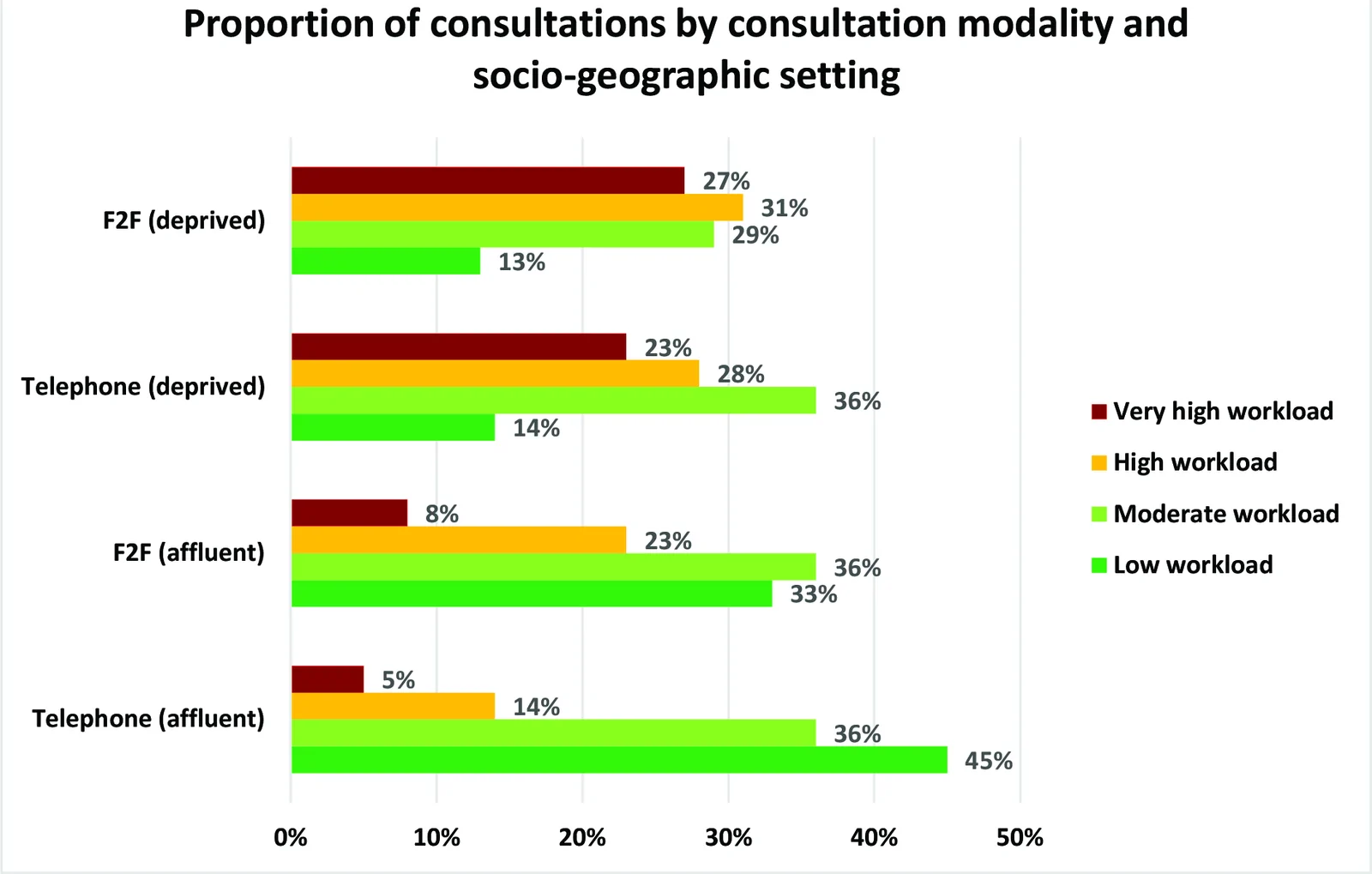
Differences in GP consultation workload by consultation modality and socio-geographic setting (affluent and deprived areas). F2F = face-to-face consultation

## Discussion

### Summary

This novel CWI integrates the following three key determinants affecting consultation workload: the presence of a disability or limiting long-term illness; the discussion of more than one problem; and the complexity of the problem(s) discussed. The weak correlations and modest overlap between these variables suggest they capture different aspects of consultation workload. Applying the CWI to consultations in Scottish general practice demonstrated consistently higher workload levels in deprived areas across all consultations, and when stratified by consultation modality (telephone and face to face). This disparity was particularly marked for telephone consultations, which showed disproportionately higher workload in deprived areas compared with affluent areas.

### Strengths and limitations

A key strength of this study is the use of patient-reported data to capture granular elements of consultation workload that are often under-recorded or omitted in electronic medical records. Previous research has shown that many problems raised by patients during consultations are not formally coded in electronic medical records, which rely on practitioner interpretation and subjectivity.^15^ This analysis also draws on a large, population-representative sample, which enhanced the generalisability of the findings.^6^

Limitations of this study include the fact that the novel index cannot easily be applied to electronic medical records, as the nature of problems (physical and/or psychosocial) is not routinely coded. In addition, while alternative variables could have been considered for the index (such as multimorbidity count), disability or limiting long-term illness was chosen as it implies a level of functional impairment, contributing to consultation complexity. Our analysis showed that disability was often present even among patients with few or no long-term conditions, supporting its inclusion (Supplementary Figures 1 and 2). This aligns with recent evidence highlighting that relying solely on chronic disease counts underestimates the multidimensional nature of patient complexity.^16^

A further possible limitation is that we did not ask the practices recruited whether they had a ‘one problem, one consultation policy’, which we know anecdotally that some practices have implemented. However, analysis of the data at practice level indicated that there were no practices in which 100% of consultations only involved one problem and all practices included patients who reported having discussed one, two, or three or more problems in their consultation.

### Comparison with existing literature

These findings contribute to the growing body of evidence on the inverse care law, which highlights the persistent mismatch between healthcare need and provision in socioeconomically deprived areas.^2,4,17^ Prior studies have shown that GPs working in deprived settings face higher demand, more complex consultations, and increased stress.^2,7^ Although national funding formulas include some adjustments for deprivation, multiple studies across the UK have demonstrated that these do not adequately account for the extent of workload variation associated with socioeconomic disadvantage in general practice.^18–21^

Measuring and defining consultation workload or complexity is challenging owing to its multifaceted nature. ^22^ Previous approaches to measuring complexity in health care have focused on hospital settings or other specialties,^23–25^ or have looked exclusively at patient-level health complexity, without consideration of other factors pertaining to the task or encounter.^26^

With respect to primary care consultation, Salisbury *et al* developed a binary complexity measure based on the presence of 17 indicators identifiable from codes in electronic medical records.^15,27,28^ However, many of these indicators — such as homelessness or safeguarding — are not routinely coded in Scottish general practice and are unlikely to be available in other contexts. Other approaches include the INTERMED tool, which measures the following four health-related aspects of complexity: (1) biological; (2) psychological; (3) social; and (4) health system. Each aspect is assessed in the context of time (history, current state, and vulnerability or prognosis) with five specific variables (items) within each domain.^29^ The information is gathered by way of semi-structured interview, and is thus not a measure that could easily be incorporated into busy routine general practice.

The CWI offers an alternative model by using patient-reported data to generate an ordinal score of workload at the consultation level, enabling a more granular assessment that moves beyond the binary approach used by Salisbury *et al.* ^28^ This approach builds on evidence that patients with disabilities consult more frequently compared to those without,^30^ and that consultations in deprived areas involve more complex and more numerous problems, compared with those in affluent areas.^2,3,31,32^

### Implications for research and practice

Current UK funding models do not capture workload at the consultation level.^33,34^ Such funding models generally use routinely available data on characteristics such as patients’ age and deprivation (based on address) with weightings applied. The allocation formula used in the new Scottish GP contract has been heavily criticised for using out-of-date data and heavily weighting age, which disproportionately rewards practices in more affluent areas where patients live to an older age.^35^ The CWI may help address these issues by offering a new consultation-level tool to quantify general practice workload pressures, which could complement existing funding metrics. This could help identify variation in consultation-level demand across socioeconomic contexts, with potential to support more equitable funding allocation, workforce distribution, and service design. Further research is warranted to formally evaluate the face and construct validity of the CWI, and explore its applicability in different healthcare contexts and record systems.

Importantly, our findings raise concerns on a previously underexplored aspect of the inverse care law, namely inequalities in telephone consultations. Our study found that telephone consultations had a disproportionately high workload in deprived areas compared with affluent areas, raising important questions about the appropriateness and safety of telephone consultations for managing patients with complex needs. Previous studies have shown that patients in deprived areas prefer face-to-face consultations, yet are more likely to receive telephone-based consultations than their counterparts in affluent areas.^6,36^ This trend may reflect policy shifts prioritising access over continuity, potentially overlooking the nature of care being delivered and the unmet needs of more complex and vulnerable patients.^37^ This study suggests that service delivery and access policies require further research to ensure they do not inadvertently exacerbate health inequalities.

Operationalising the CWI from patient-reported data would be challenging. However, advances in real-time AI may offer promising avenues for future application. Emerging AI systems are increasingly capable of extracting relevant clinical information from electronic health records and generating live consultation summaries within consultations.^38,39^ Such technologies could enable automated detection of the number of problems discussed and complexity during consultations, while disability may be inferred from health records or proxied by a high number of long-term conditions. These approaches merit further investigation to capture consultation-level complexity and inform equitable resource allocation.

In conclusion, the CWI provides a novel, patient-reported measure of workload at the consultation level, demonstrating significant socioeconomic disparities in workload, particularly for telephone consultations in deprived areas. These findings highlight limitations of current general practice funding models in the UK and underscore the need for tools that better reflect the true burden of general practice in deprived areas. Wider use of tools, such as the CWI, could support more equitable service delivery and contribute to addressing the persistent effects of the inverse care law.

## Data

The authors do not have ethical permission or patient consent to share the full data.
